# Synthesis and crystal structure of ethyl 2-{[4-amino-5-cyano-6-(3,4-di­meth­oxy­phen­yl)pyrimidin-2-yl]sulfan­yl}acetate

**DOI:** 10.1107/S2056989026010352

**Published:** 2026-10-20

**Authors:** Manar M. Abuzahra, Wafaa A. Zaghary, Nesreen S. Ahmed, Mohammed A. Khedr, Hesham R. S. Haffez, Galal H. Elgemeie, Peter G. Jones

**Affiliations:** a Pharmaceutical Chemistry Deapartment, Faculty of Pharmacy, Capital University, Helwan, Egypt; b Therapeutic Chemistry Department, National Research Centre, Dokki, Cairo, Egypt; c Biochemistry and Molecular Biology, Faculty of Pharmacy, Capital University, Helwan, Egypt; d Chemistry Department, Faculty of Science, Capital University, Helwan, Egypt; e Institut für Anorganische und Analytische Chemie, Technische Universität Braunschweig, Hagenring 30, D-38106 Braunschweig, Germany; Universität Greifswald, Germany

**Keywords:** crystal structure, pyrimidine, hydrogen bond, ester, acetate

## Abstract

In the title com­pound, C_17_H_18_N_4_O_4_S, the pyrimidine and phenyl rings subtend an inter­planar angle of 30.05 (5)°. The ring angle at the C atom between the N atoms is very wide at 129.32 (11)° and the angles at the ring N atoms are narrow [115.58 (10) and 115.98 (10)°]. The mol­ecules are linked by two classical hy­dro­gen bonds with the amine group as donor, namely N—H⋯N≡C and N—H⋯O=C, *via* an inversion centre and a 2_1_ screw axis, respectively. There are also short contacts of 3.6006 (7) Å between the centroids of pairs of pyrimidine rings across inversion centres.

## Abstract 2

In the title com­pound, the pyrimidine ring angle at the C atom between the N atoms is very wide, whereas the angles at the ring N atoms are narrow. The mol­ecules are linked by N—H⋯N≡C and N—H⋯O=C hy­dro­gen bonds, and by short inter­centroid contacts between pairs of pyrimidine rings.

## 1. Chemical context

In medicinal chemistry, pyrimidines are an important class of nitro­gen-containing heterocycles. As com­ponents of nucleic acids, they are necessarily constituents of every cell, and thus of all biological living matter. They are also present in many other naturally occurring mol­ecules, such as vitamins, coenzymes and uric acids. Various pyrimidine scaffolds have been designed and used by chemists to produce innovative medicaments with a range of pharmacological activities (Luo *et al.*, 2026 ▸). There are many clinically approved drugs containing pyrimidine in their structures; some examples are 5-fluoro­uracil and Gemcitabine as anti­cancer, Zidovudine and Stavudine as anti-HIV, Bu­spirone as anti-anxiety, and Trimethoprim, Sulfadiazine and Sulfadoxine as anti­microbial agents (Sahu *et al.*, 2016 ▸; Stolarczyk *et al.*, 2018 ▸). One significant family of pyrimidines consists of 2-thio­pyrimidine (2-TP) and its derivatives, also well known as 2-mercapto­pyrimidine com­pounds, in which the S atom replaces the O atom of uridine-based systems. 2-TP derivatives have found a wide range of applications in the preparation of cardiotonic drugs and anticancer, antitubercular, anti-inflammatory and anti­bacterial agents (Lamie *et al.*, 2020 ▸; El-serwy *et al.*, 2019 ▸). They have attracted the attention of chemists and biochemists who wish to develop the range of potentially therapeutic com­pounds bearing thio­pyrimidine scaffolds (Sondhi *et al.*, 2005 ▸; Rabeh *et al.*, 2026 ▸).

We have recently described various synthetic approaches to the synthesis of purine and pyrimidine analogs (Abu-Zaied *et al.*, 2020 ▸; Abu-Zaied *et al.*, 2021 ▸). In the majority of biochemical reactions, several derivatives of these ring structures are regarded as crucial anti­metabolites (Khedr *et al.*, 2022 ▸; Khedr *et al.*, 2023 ▸). As a continuation of these studies of the synthesis of biologically active anti­metabolic agents (Elgemeie *et al.*, 2019 ▸; Elgemeie *et al.*, 2022 ▸), the current study reports the synthesis of ethyl 2-{[4-amino-5-cyano-6-(3,4-di­meth­oxy­phen­yl)pyrimidin-2-yl]sulfan­yl}acetate, **4**, by re­ac­ting the pyrimidine-2-thiol derivative **3** with ethyl chloro­acetate (Fig. 1 ▸). The crystal structure was determined to confirm the nature of **4**, and is reported here.
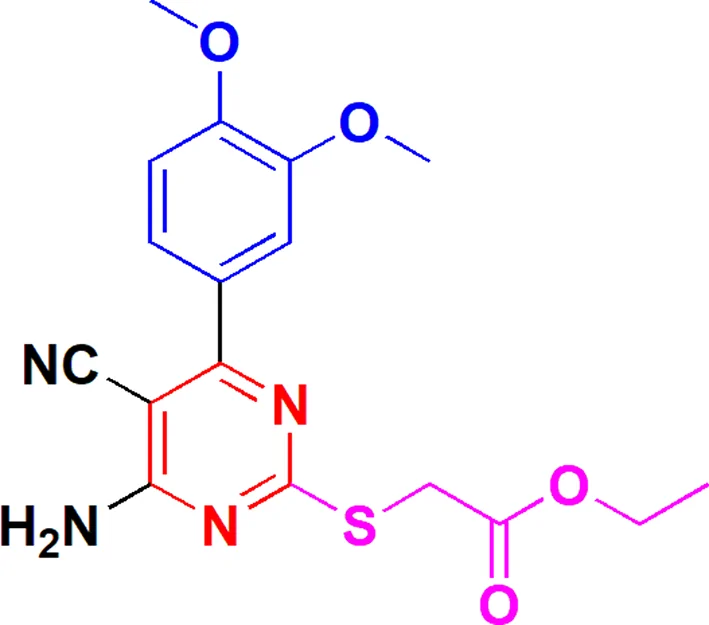


## 2. Structural commentary

The mol­ecule of com­pound **4** in the crystal is shown in Fig. 2 ▸, with selected mol­ecular dimensions in Table 1 ▸. Both rings are essentially planar, with r.m.s. deviations of 0.010 and 0.013 Å for the pyrimidine and phenyl ring, respectively. The only substituent lying significantly out-of-plane is C7 [by 0.221 (2) Å]. The inter­planar angle is 30.05 (5)°. The ring angle at C2 is the widest at 129.32 (11)°, associated with a narrow angle N3—C2—S1 of 112.01 (8)° at the planar centre C2. The narrowest ring angles are those at the N atoms [115.58 (10) and 115.98 (10)°]. The bond length S1—C2 is significantly shorter than S1—C8, consistent with the different hybridizations of the C atoms; similar considerations apply to C5—C7 being shorter than C6—C21. The NH_2_ group is planar, with the N atom lying an insignificant 0.003 (9) Å out of the plane of its substituents. The atom sequence C8—C9—O2—C10—C11 of the side chain displays an extended conformation.

## 3. Supra­molecular features

The main inter­molecular contacts are the classical hy­dro­gen bonds from the H atoms of the amino group, H021 and H022, to the acceptors N4 and O1 of adjacent mol­ecules, *via* an inversion centre and the 2_1_ screw axis, respectively (Table 2 ▸, which also includes some borderline weak hy­dro­gen bonds). Fig. 3 ▸ shows the infinite chains of mol­ecules parallel to the *b* axis formed by the contact H022⋯O1. It also shows (implicitly) the short contacts between pairs of pyrimidine rings connected by inversion, with a centroid-to-centroid distance of 3.6006 (7) Å and an offset of 1.2 Å (symmetry operator: −*x* + 2, −*y* + 1, −*z* + 1) for the pair involving the reference mol­ecule.

The combination of inversion and 2_1_ symmetry for the two hy­dro­gen bonds means that the overall packing is three-dimensional rather than a ribbon or layer structure. The second packing diagram (Fig. 4 ▸) is therefore a projection showing the combination of both hy­dro­gen bonds.

## 4. Database survey

Searches were conducted using the Cambridge Structural Database (CSD, Version 6.01, update November 2025; Groom *et al.*, 2016 ▸) and the *ConQuest* routine (Version 2025.3.1; Bruno *et al.*, 2002 ▸), and were restricted to non-disordered ‘organic’ structures.

A general search for pyrimidine systems with no further annelation gave 2950 hits. The angle at C2 was consistently and significantly larger than 120°, with a mean angle of 126.9 (17)° for 3782 values. A search restricted to the substitution pattern of com­pound **4** (S at C2, N at C4, CN at C5 and C at C6) gave just two hits: 4-[4-(7-chloro­quino­lin-4-yl)pi­per­a­zin-1-yl]-2-(methyl­sul­fan­yl)-6-phenyl­py­rimi­dine-5-car­bo­ni­trile (refcode HUKRUC; Kaur *et al.*, 2015 ▸) and 2-[(4-chloro­benz­yl)sulfan­yl]-4-(2-methyl­prop­yl)-6-[3-(tri­fluoro­meth­yl)anil­ino]py­rimi­dine-5-car­bo­ni­trile (XAZLUH; El-Emam *et al.*, 2012 ▸). However, neither of these can be regarded as closely similar to **4**, thus underlining its novel nature.

## 5. Synthesis and crystallization

A mixture of com­pound **3** (1 mmol) and ethyl chloro­acetate (1.2 mmol) in aceto­nitrile containing a few drops of tri­ethyl­amine was stirred at room tem­per­a­ture for 48 h. The precipitate thus formed was filtered off and washed with ethanol to give the desired com­pound **4**, as a buff powder, which was recrystallized from aceto­nitrile to give pale-yellow crystals (isolated single crystals are effectively colourless) in 65% yield.

M.p. 453–455 K; IR (KBr, ν cm^−1^): 3470, 3347 (NH_2_) 2206 (CN), 1736, (C=O); ^1^H NMR (DMSO-*d*_6_, 500 MHz): δ (ppm) 1.11 (*t*, *J* = 7.0 Hz, 3H, CH_2_C*H*_3_), 3.84 (*s*, 3H, OCH_3_), 3.85 (*s*, 3H, OCH_3_), 4.03 (*s*, 2H, S-C*H*_2_), 4.07 (*q*, *J* = 7.0 Hz, 2H, C*H*_2_CH_3_), 7.12 (*d*, *J* = 8.5 Hz, 1H, Ar-H), 7.54 (*d*, *J* = 2.0 Hz, 1H, Ar-H), 7.57 (*d*, *J* = 8.5 Hz, 1H, Ar-H), 8.03 (*br s*, 2H, NH_2_, D_2_O-exchangeable); ^13^C NMR (DMSO-*d_6_*, 150 MHz): δ (ppm) 14.4 (CH_2_CH_3_), 33.5 (S—CH_2_), 56.2 (2OCH_3_), 61.6 (CH_2_CH_3_), 82.3, 111.6, 112.4, 117.1, 122.6, 128.2, 148.8, 152.0, 164.1, 166.7, 169.4 (C=O ester), 172.4. Analysis calculated (%) for C_17_H_18_N_4_O_4_S: C 54.53, H 4.85, N 14.96, S 8.56; found: C 54.54, H 5.13, N 14.85, S 8.74.

## 6. Refinement

Details of data collection and structure refinement are summarized in Table 3 ▸. H atoms of the NH_2_ group were refined freely. Methyl groups were refined as idealized rigid groups allowed to rotate but not tip, with C—H = 0.98 Å and H—C—H = 109.5°. Other H atoms were refined using a riding model, with aromatic C—H = 0.95 Å and methyl­ene C—H = 0.99 Å. The *U*_iso_(H) values were fixed at 1.5*U*_eq_ of the parent C atoms for the methyl groups and 1.2*U*_eq_ for the other H atoms. Two badly fitting reflections (Δ/σ > 8) were omitted from the refinement.

## Supplementary Material

Crystal structure: contains datablock(s) I, global. DOI: 10.1107/S2056989026010352/yz2085sup1.cif

Structure factors: contains datablock(s) I. DOI: 10.1107/S2056989026010352/yz2085Isup2.hkl

Supporting information file. DOI: 10.1107/S2056989026010352/yz2085Isup3.cml

CCDC reference: 2593312

Additional supporting information:  crystallographic information; 3D view; checkCIF report

## Figures and Tables

**Figure 1:**
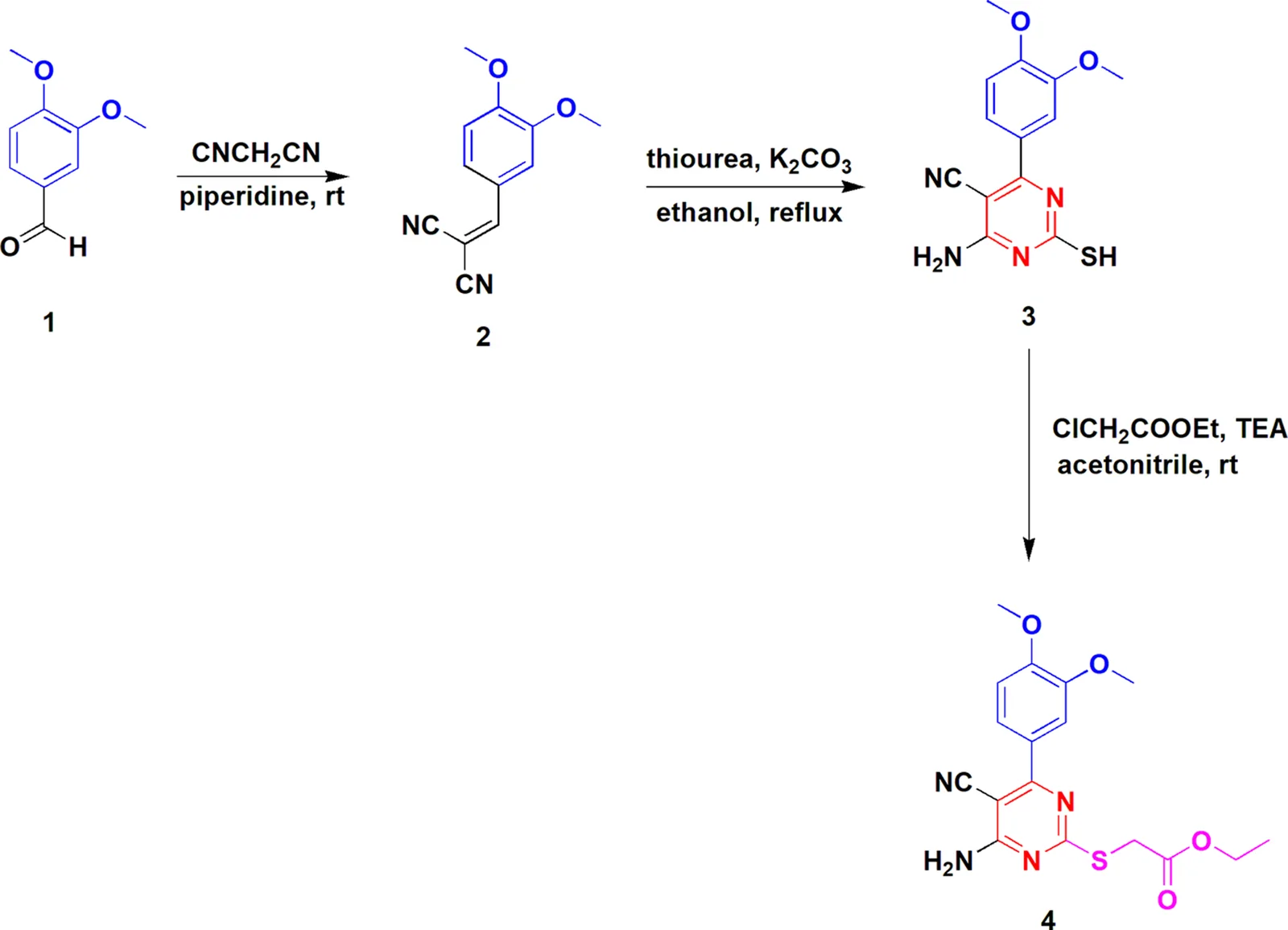
The synthesis of com­pound **4**.

**Figure 2:**
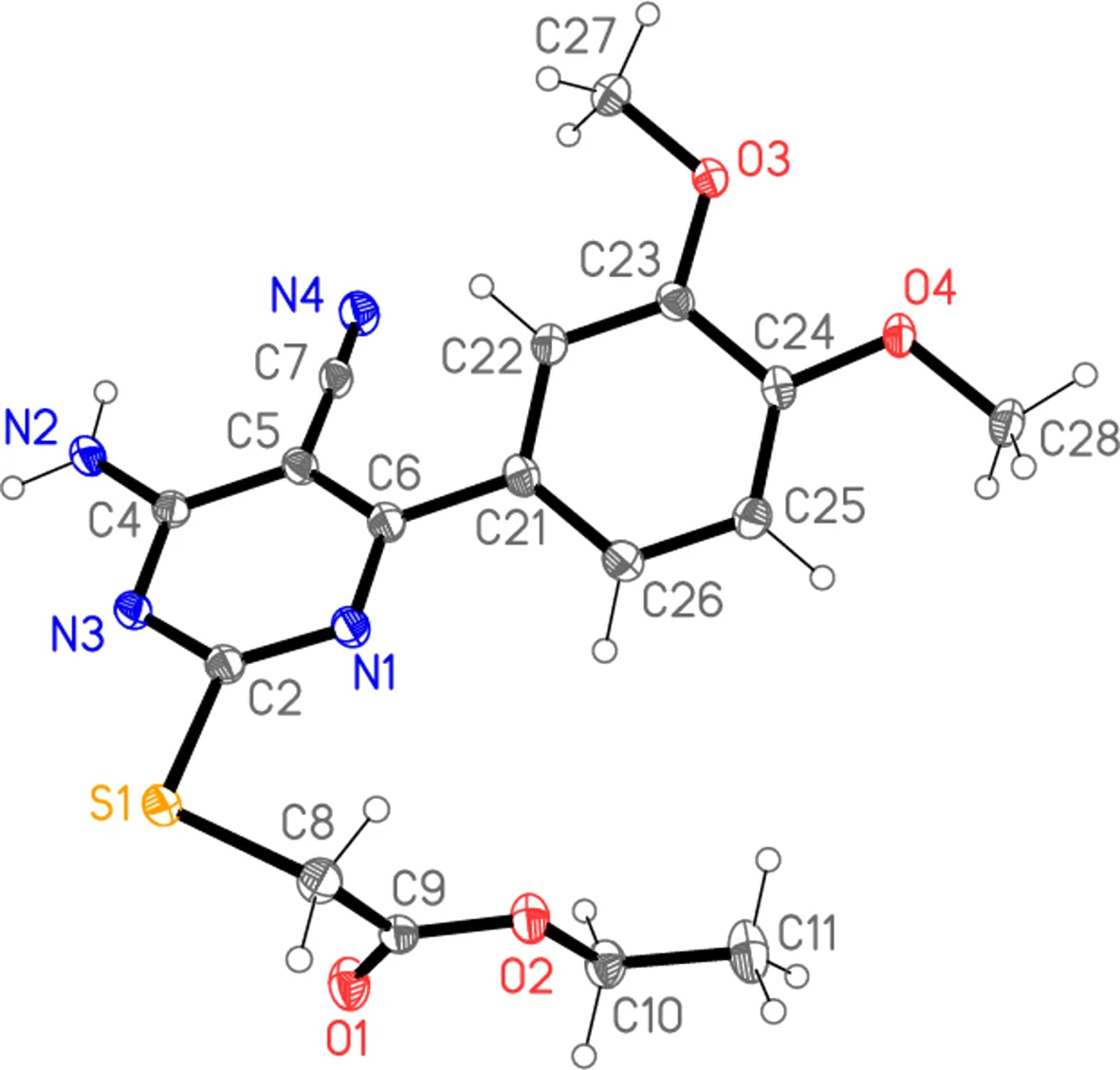
The mol­ecule of com­pound **4** in the crystal. Displacement ellipsoids represent 50% probability levels.

**Figure 3:**
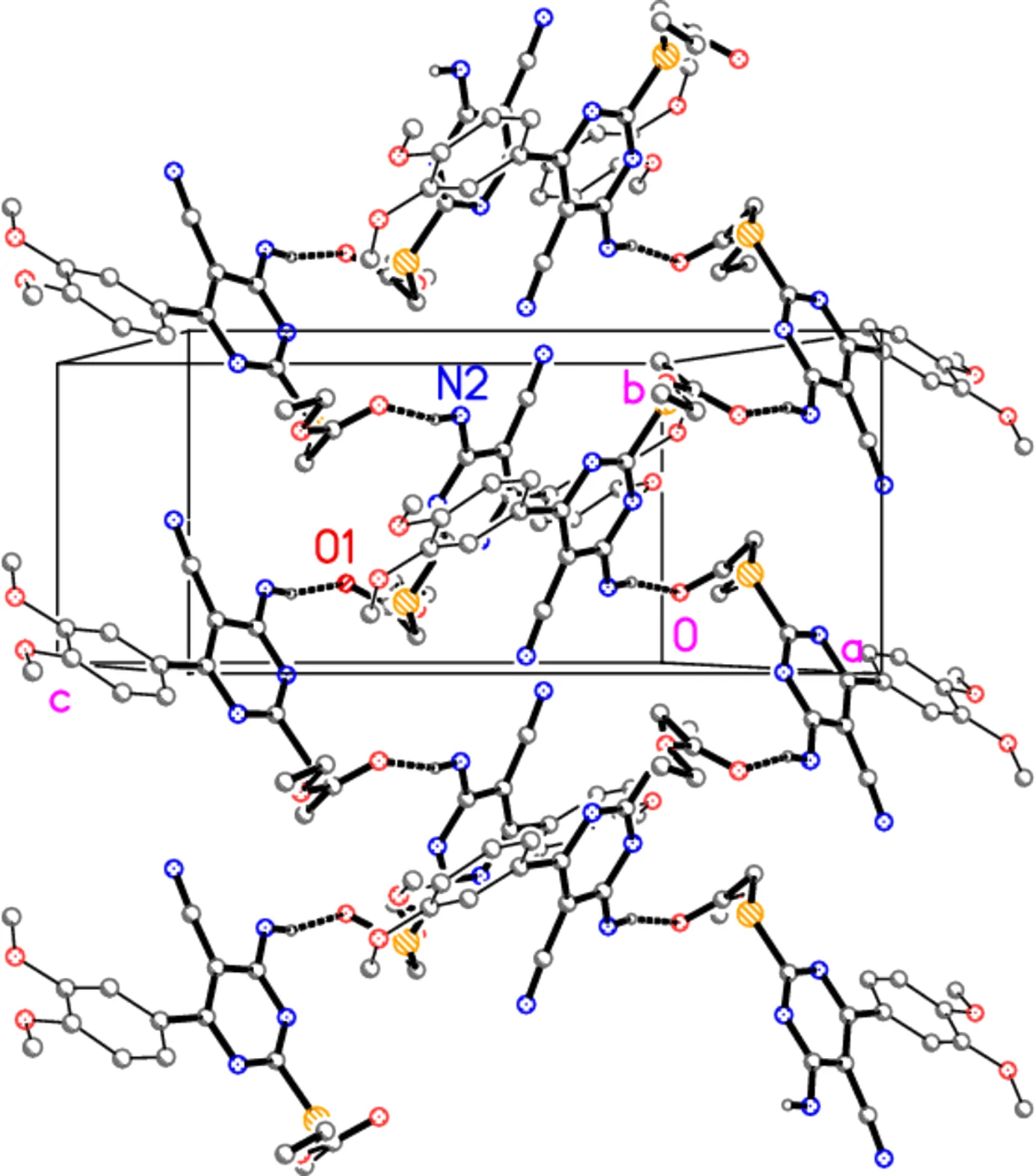
Two chains of mol­ecules of com­pound **4**, running vertically, linked by the hy­dro­gen bonds N2⋯H022—O1 (thick dashed lines). The view direction is perpendicular to the *bc* plane, in the region *a* ≃ 0.75. Atom names indicate the asymmetric unit. H atoms not involved in these interactions are omitted for clarity.

**Figure 4:**
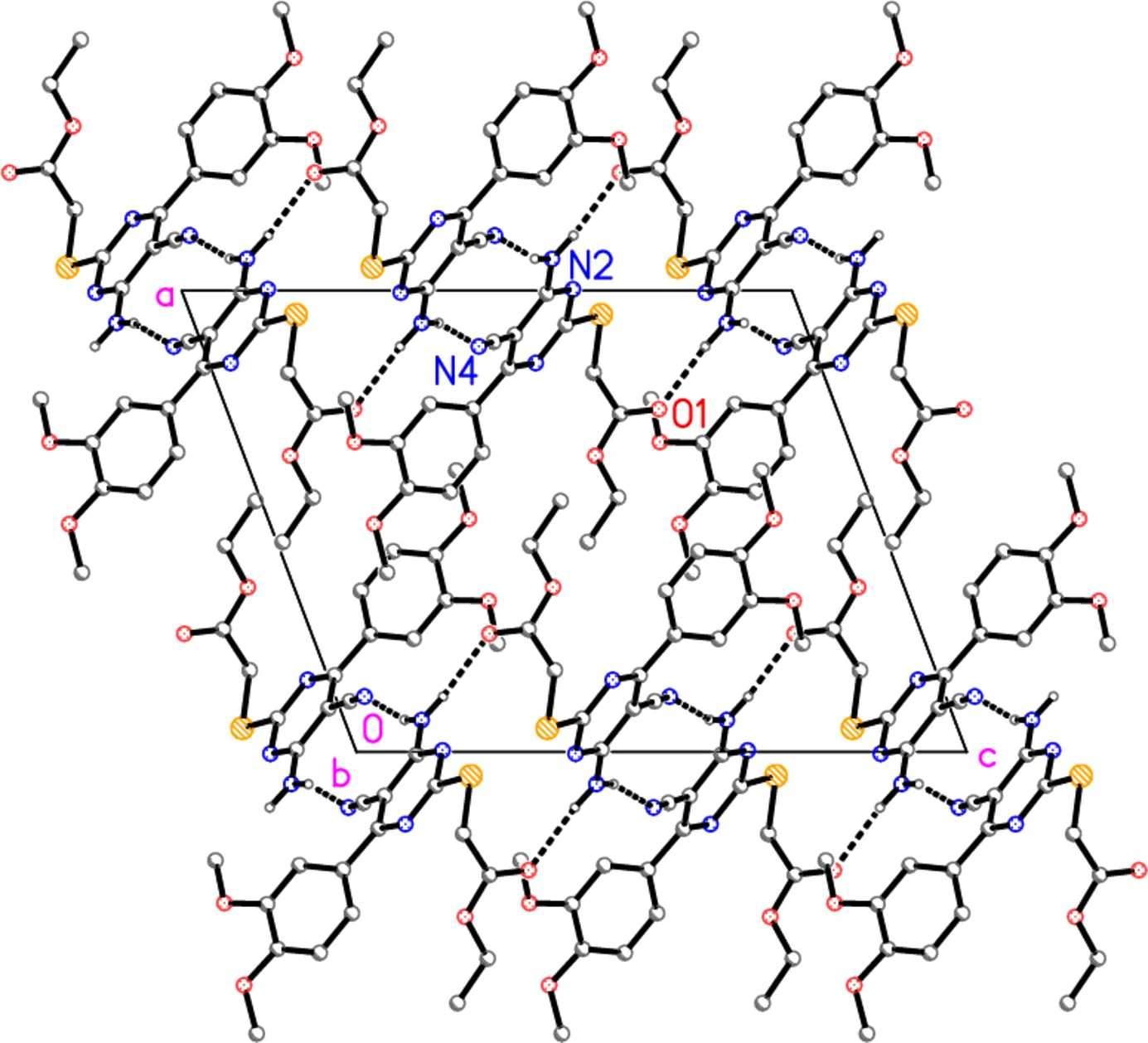
A projection of the three-dimensional packing of com­pound **4** parallel to the *b* axis, showing linkages *via* both classical hy­dro­gen bonds (thick dashed lines). Atom names indicate the asymmetric unit. H atoms not involved in these interactions are omitted for clarity.

**Table 1:** Selected geometric parameters (Å, °)

S1—C2	1.7619 (12)	N3—C4	1.3473 (15)
S1—C8	1.7987 (12)	C4—C5	1.4335 (15)
N1—C2	1.3348 (14)	C5—C6	1.3980 (16)
N1—C6	1.3508 (15)	C5—C7	1.4250 (16)
C2—N3	1.3323 (15)	C6—C21	1.4801 (15)
			
C2—S1—C8	101.07 (6)	N3—C4—C5	119.76 (10)
C2—N1—C6	115.58 (10)	C6—C5—C4	118.35 (10)
N3—C2—N1	129.32 (11)	N1—C6—C5	120.76 (10)
C2—N3—C4	115.98 (10)		
			
C8—S1—C2—N1	−13.64 (10)	C8—C9—O2—C10	−179.59 (9)
C2—S1—C8—C9	−69.51 (9)	C11—C10—O2—C9	−174.39 (10)
S1—C8—C9—O2	144.75 (8)		

**Table 2:** Hydrogen-bond geometry (Å, °)

*D*—H⋯*A*	*D*—H	H⋯*A*	*D*⋯*A*	*D*—H⋯*A*
N2—H021⋯N4^i^	0.862 (19)	2.201 (19)	3.0368 (15)	163.4 (16)
N2—H022⋯O1^ii^	0.879 (19)	2.139 (19)	2.9942 (13)	164.0 (16)
C8—H8*A*⋯N4^iii^	0.99	2.67	3.4693 (16)	138
C11—H11*A*⋯O3^iv^	0.98	2.52	3.1459 (15)	122
C27—H27*B*⋯S1^v^	0.98	2.95	3.6836 (12)	133

Symmetry codes: (i) 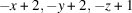; (ii) 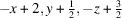; (iii) 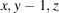; (iv) 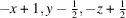; (v) 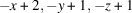.

**Table 3:** Experimental details

Crystal data	
Chemical formula	C_17_H_18_N_4_O_4_S
*M*_r_	374.41
Crystal system, space group	Monoclinic, *P*2_1_/*c*
Temperature (K)	100
*a*, *b*, *c* (Å)	13.4276 (5), 8.2128 (3), 16.6201 (7)
β (°)	110.762 (5)
*V* (Å^3^)	1713.80 (13)
*Z*	4
Radiation type	Cu *K*α
μ (mm^−1^)	1.97
Crystal size (mm)	0.14 × 0.05 × 0.03

Data collection	
Diffractometer	Rigaku XtaLAB Synergy
Absorption correction	Multi-scan (*CrysAlis PRO*; Rigaku OD, 2026 ▸)
*T*_min_, *T*_max_	0.763, 1.000
No. of measured, independent and observed [*I* > 2σ(*I*)] reflections	78100, 3747, 3512
*R*_int_	0.044
θ values (°)	θ_max_ = 81.6, θ_min_ = 3.5
(sin θ/λ)_max_ (Å^−1^)	0.642

Refinement	
*R*[*F*^2^ > 2σ(*F*^2^)], *wR*(*F*^2^), *S*	0.030, 0.079, 1.09
No. of reflections	3747
No. of parameters	246
H-atom treatment	H atoms treated by a mixture of independent and constrained refinement
Δρ_max_, Δρ_min_ (e Å^−3^)	0.33, −0.27

Computer programs: *CrysAlis PRO* (Rigaku OD, 2026 ▸), *SHELXT* (Sheldrick, 2015*a* ▸), *SHELXL2019* (Sheldrick, 2015*b* ▸), *XP* (Bruker, 1998 ▸) and *publCIF* (Westrip, 2010 ▸).

## supplementary crystallographic information

### Ethyl 2-{[4-amino-5-cyano-6-(3,4-dimethoxyphenyl)pyrimidin-2-yl]sulfanyl}acetate Crystal data

**Table:**

C_17_H_18_N_4_O_4_S	*F*(000) = 784
*M**_r_* = 374.41	*D*_x_ = 1.451 Mg m^−^^3^
Monoclinic, *P*2_1_/*c*	Cu *K*α radiation, λ = 1.54184 Å
*a* = 13.4276 (5) Å	Cell parameters from 44633 reflections
*b* = 8.2128 (3) Å	θ = 3.5–80.1°
*c* = 16.6201 (7) Å	µ = 1.97 mm^−^^1^
β = 110.762 (5)°	*T* = 100 K
*V* = 1713.80 (13) Å^3^	Prism, colourless
*Z* = 4	0.14 × 0.05 × 0.03 mm

### Ethyl 2-{[4-amino-5-cyano-6-(3,4-dimethoxyphenyl)pyrimidin-2-yl]sulfanyl}acetate Data collection

**Table:**

Rigaku XtaLAB Synergy diffractometer	3747 independent reflections
Radiation source: micro-focus sealed X-ray tube, PhotonJet (Cu) X-ray Source	3512 reflections with *I* > 2σ(*I*)
Mirror monochromator	*R*_int_ = 0.044
Detector resolution: 10.0000 pixels mm^-1^	θ_max_ = 81.6°, θ_min_ = 3.5°
ω scans	*h* = −16→17
Absorption correction: multi-scan (CrysAlis PRO; Rigaku OD, 2026)	*k* = −10→10
*T*_min_ = 0.763, *T*_max_ = 1.000	*l* = −21→21
78100 measured reflections	

### Ethyl 2-{[4-amino-5-cyano-6-(3,4-dimethoxyphenyl)pyrimidin-2-yl]sulfanyl}acetate Refinement

**Table:**

Refinement on *F*^2^	Primary atom site location: dual
Least-squares matrix: full	Hydrogen site location: mixed
*R*[*F*^2^ > 2σ(*F*^2^)] = 0.030	H atoms treated by a mixture of independent and constrained refinement
*wR*(*F*^2^) = 0.079	*w* = 1/[σ^2^(*F*_o_^2^) + (0.038*P*)^2^ + 0.7704*P*] where *P* = (*F*_o_^2^ + 2*F*_c_^2^)/3
*S* = 1.09	(Δ/σ)_max_ = 0.001
3747 reflections	Δρ_max_ = 0.33 e Å^−^^3^
246 parameters	Δρ_min_ = −0.27 e Å^−^^3^
0 restraints	

### Ethyl 2-{[4-amino-5-cyano-6-(3,4-dimethoxyphenyl)pyrimidin-2-yl]sulfanyl}acetate Fractional atomic coordinates and isotropic or equivalent isotropic displacement parameters (Å^2^)

**Table:**

	*x*	*y*	*z*	*U*_iso_*/*U*_eq_	
S1	0.94829 (2)	0.20843 (4)	0.67241 (2)	0.01892 (9)	
N1	0.84359 (8)	0.39055 (12)	0.53524 (6)	0.0170 (2)	
C2	0.92716 (9)	0.38561 (14)	0.60876 (7)	0.0165 (2)	
N3	1.00361 (7)	0.49571 (12)	0.64206 (6)	0.0171 (2)	
C4	0.99444 (9)	0.63454 (14)	0.59681 (7)	0.0167 (2)	
C5	0.90500 (9)	0.65719 (14)	0.51876 (7)	0.0162 (2)	
C6	0.83380 (9)	0.52791 (14)	0.48834 (7)	0.0160 (2)	
C7	0.88980 (9)	0.81410 (14)	0.47967 (7)	0.0172 (2)	
C8	0.82267 (9)	0.10613 (14)	0.62413 (8)	0.0194 (2)	
H8A	0.805412	0.098954	0.561170	0.023*	
H8B	0.828418	−0.006128	0.647040	0.023*	
C9	0.73379 (9)	0.19453 (14)	0.64200 (7)	0.0167 (2)	
C10	0.55229 (9)	0.26761 (15)	0.58683 (8)	0.0209 (2)	
H10A	0.564619	0.386653	0.591305	0.025*	
H10B	0.542433	0.229385	0.640011	0.025*	
C11	0.45556 (10)	0.22845 (19)	0.50957 (8)	0.0292 (3)	
H11A	0.467709	0.261922	0.457205	0.044*	
H11B	0.393828	0.286890	0.513533	0.044*	
H11C	0.442029	0.110989	0.507580	0.044*	
C21	0.74471 (9)	0.53373 (14)	0.40457 (7)	0.0161 (2)	
C22	0.75448 (9)	0.62478 (14)	0.33607 (7)	0.0159 (2)	
H22	0.819795	0.677987	0.342878	0.019*	
C23	0.67021 (9)	0.63755 (14)	0.25914 (7)	0.0160 (2)	
C24	0.57304 (9)	0.55837 (13)	0.24843 (7)	0.0156 (2)	
C25	0.56457 (9)	0.46370 (14)	0.31507 (7)	0.0177 (2)	
H25	0.500013	0.407833	0.307827	0.021*	
C26	0.65032 (9)	0.45039 (14)	0.39242 (7)	0.0179 (2)	
H26	0.644264	0.383860	0.437216	0.021*	
C27	0.76480 (10)	0.82281 (16)	0.20173 (8)	0.0217 (2)	
H27A	0.755218	0.884562	0.149059	0.033*	
H27B	0.827392	0.752223	0.214699	0.033*	
H27C	0.775101	0.898458	0.249615	0.033*	
C28	0.38928 (9)	0.52854 (16)	0.16163 (8)	0.0225 (2)	
H28A	0.338779	0.564480	0.105858	0.034*	
H28B	0.368932	0.574936	0.207907	0.034*	
H28C	0.388298	0.409430	0.164783	0.034*	
O1	0.74353 (7)	0.26372 (12)	0.70858 (5)	0.02394 (19)	
O2	0.64265 (6)	0.18454 (11)	0.57556 (5)	0.02001 (18)	
O3	0.67237 (6)	0.72523 (11)	0.18981 (5)	0.02081 (19)	
O4	0.49428 (6)	0.58233 (10)	0.17089 (5)	0.01874 (18)	
N2	1.06917 (8)	0.74770 (14)	0.62655 (7)	0.0207 (2)	
H021	1.0695 (14)	0.837 (2)	0.5990 (11)	0.033 (4)*	
H022	1.1204 (14)	0.733 (2)	0.6765 (12)	0.030 (4)*	
N4	0.87969 (8)	0.94458 (13)	0.45245 (6)	0.0220 (2)	

### Ethyl 2-{[4-amino-5-cyano-6-(3,4-dimethoxyphenyl)pyrimidin-2-yl]sulfanyl}acetate Atomic displacement parameters (Å^2^)

**Table:**

	*U*^11^	*U*^22^	*U*^33^	*U*^12^	*U*^13^	*U*^23^
S1	0.01518 (14)	0.02360 (15)	0.01707 (14)	0.00250 (10)	0.00460 (11)	0.00545 (10)
N1	0.0160 (4)	0.0198 (5)	0.0144 (4)	0.0012 (4)	0.0043 (4)	0.0008 (4)
C2	0.0156 (5)	0.0214 (6)	0.0139 (5)	0.0034 (4)	0.0068 (4)	0.0004 (4)
N3	0.0150 (4)	0.0231 (5)	0.0133 (4)	0.0014 (4)	0.0052 (3)	0.0012 (4)
C4	0.0159 (5)	0.0226 (6)	0.0124 (5)	0.0005 (4)	0.0062 (4)	−0.0016 (4)
C5	0.0152 (5)	0.0202 (5)	0.0130 (5)	0.0020 (4)	0.0049 (4)	0.0000 (4)
C6	0.0150 (5)	0.0191 (5)	0.0139 (5)	0.0030 (4)	0.0052 (4)	−0.0009 (4)
C7	0.0138 (5)	0.0232 (6)	0.0133 (5)	−0.0011 (4)	0.0032 (4)	−0.0037 (4)
C8	0.0190 (5)	0.0182 (5)	0.0208 (5)	0.0002 (4)	0.0069 (4)	0.0003 (4)
C9	0.0165 (5)	0.0165 (5)	0.0162 (5)	−0.0019 (4)	0.0046 (4)	0.0021 (4)
C10	0.0173 (6)	0.0254 (6)	0.0194 (5)	0.0023 (5)	0.0058 (5)	−0.0026 (5)
C11	0.0200 (6)	0.0403 (8)	0.0224 (6)	0.0054 (5)	0.0014 (5)	−0.0069 (5)
C21	0.0159 (5)	0.0162 (5)	0.0147 (5)	0.0024 (4)	0.0036 (4)	−0.0011 (4)
C22	0.0137 (5)	0.0175 (5)	0.0156 (5)	0.0004 (4)	0.0040 (4)	−0.0017 (4)
C23	0.0164 (5)	0.0172 (5)	0.0141 (5)	0.0010 (4)	0.0049 (4)	−0.0007 (4)
C24	0.0142 (5)	0.0157 (5)	0.0145 (5)	0.0012 (4)	0.0020 (4)	−0.0020 (4)
C25	0.0165 (5)	0.0165 (5)	0.0193 (5)	−0.0018 (4)	0.0052 (4)	−0.0013 (4)
C26	0.0199 (5)	0.0173 (5)	0.0157 (5)	0.0000 (4)	0.0052 (4)	0.0005 (4)
C27	0.0175 (5)	0.0256 (6)	0.0211 (6)	−0.0054 (5)	0.0056 (4)	0.0014 (5)
C28	0.0131 (5)	0.0272 (6)	0.0232 (6)	−0.0034 (5)	0.0015 (4)	0.0016 (5)
O1	0.0190 (4)	0.0330 (5)	0.0179 (4)	0.0016 (4)	0.0042 (3)	−0.0049 (4)
O2	0.0161 (4)	0.0250 (4)	0.0172 (4)	0.0013 (3)	0.0038 (3)	−0.0031 (3)
O3	0.0166 (4)	0.0282 (5)	0.0146 (4)	−0.0054 (3)	0.0019 (3)	0.0041 (3)
O4	0.0131 (4)	0.0228 (4)	0.0162 (4)	−0.0025 (3)	0.0002 (3)	0.0016 (3)
N2	0.0183 (5)	0.0260 (5)	0.0139 (5)	−0.0042 (4)	0.0011 (4)	0.0019 (4)
N4	0.0216 (5)	0.0219 (5)	0.0198 (5)	−0.0010 (4)	0.0040 (4)	0.0001 (4)

### Ethyl 2-{[4-amino-5-cyano-6-(3,4-dimethoxyphenyl)pyrimidin-2-yl]sulfanyl}acetate Geometric parameters (Å, º)

**Table:**

S1—C2	1.7619 (12)	C11—H11C	0.9800
S1—C8	1.7987 (12)	C21—C26	1.3908 (16)
N1—C2	1.3348 (14)	C21—C22	1.4066 (16)
N1—C6	1.3508 (15)	C22—C23	1.3786 (15)
C2—N3	1.3323 (15)	C22—H22	0.9500
N3—C4	1.3473 (15)	C23—O3	1.3677 (13)
C4—N2	1.3279 (16)	C23—C24	1.4115 (16)
C4—C5	1.4335 (15)	C24—O4	1.3609 (13)
C5—C6	1.3980 (16)	C24—C25	1.3905 (16)
C5—C7	1.4250 (16)	C25—C26	1.3930 (16)
C6—C21	1.4801 (15)	C25—H25	0.9500
C7—N4	1.1522 (16)	C26—H26	0.9500
C8—C9	1.5136 (16)	C27—O3	1.4309 (14)
C8—H8A	0.9900	C27—H27A	0.9800
C8—H8B	0.9900	C27—H27B	0.9800
C9—O1	1.2091 (15)	C27—H27C	0.9800
C9—O2	1.3288 (14)	C28—O4	1.4328 (14)
C10—O2	1.4608 (14)	C28—H28A	0.9800
C10—C11	1.5023 (17)	C28—H28B	0.9800
C10—H10A	0.9900	C28—H28C	0.9800
C10—H10B	0.9900	N2—H021	0.862 (19)
C11—H11A	0.9800	N2—H022	0.879 (19)
C11—H11B	0.9800		
			
C2—S1—C8	101.07 (6)	C26—C21—C22	119.06 (10)
C2—N1—C6	115.58 (10)	C26—C21—C6	120.78 (10)
N3—C2—N1	129.32 (11)	C22—C21—C6	120.16 (10)
N3—C2—S1	112.01 (8)	C23—C22—C21	120.65 (10)
N1—C2—S1	118.62 (9)	C23—C22—H22	119.7
C2—N3—C4	115.98 (10)	C21—C22—H22	119.7
N2—C4—N3	118.49 (10)	O3—C23—C22	124.46 (10)
N2—C4—C5	121.75 (11)	O3—C23—C24	115.54 (10)
N3—C4—C5	119.76 (10)	C22—C23—C24	120.00 (10)
C6—C5—C7	123.56 (10)	O4—C24—C25	125.43 (10)
C6—C5—C4	118.35 (10)	O4—C24—C23	115.22 (10)
C7—C5—C4	117.94 (10)	C25—C24—C23	119.35 (10)
N1—C6—C5	120.76 (10)	C24—C25—C26	120.31 (10)
N1—C6—C21	116.78 (10)	C24—C25—H25	119.8
C5—C6—C21	122.46 (10)	C26—C25—H25	119.8
N4—C7—C5	176.29 (12)	C21—C26—C25	120.53 (10)
C9—C8—S1	111.62 (8)	C21—C26—H26	119.7
C9—C8—H8A	109.3	C25—C26—H26	119.7
S1—C8—H8A	109.3	O3—C27—H27A	109.5
C9—C8—H8B	109.3	O3—C27—H27B	109.5
S1—C8—H8B	109.3	H27A—C27—H27B	109.5
H8A—C8—H8B	108.0	O3—C27—H27C	109.5
O1—C9—O2	123.95 (11)	H27A—C27—H27C	109.5
O1—C9—C8	124.61 (10)	H27B—C27—H27C	109.5
O2—C9—C8	111.43 (10)	O4—C28—H28A	109.5
O2—C10—C11	107.23 (10)	O4—C28—H28B	109.5
O2—C10—H10A	110.3	H28A—C28—H28B	109.5
C11—C10—H10A	110.3	O4—C28—H28C	109.5
O2—C10—H10B	110.3	H28A—C28—H28C	109.5
C11—C10—H10B	110.3	H28B—C28—H28C	109.5
H10A—C10—H10B	108.5	C9—O2—C10	115.26 (9)
C10—C11—H11A	109.5	C23—O3—C27	117.04 (9)
C10—C11—H11B	109.5	C24—O4—C28	117.51 (9)
H11A—C11—H11B	109.5	C4—N2—H021	122.7 (12)
C10—C11—H11C	109.5	C4—N2—H022	119.3 (11)
H11A—C11—H11C	109.5	H021—N2—H022	118.1 (16)
H11B—C11—H11C	109.5		
			
C6—N1—C2—N3	−1.24 (17)	N1—C6—C21—C22	−149.74 (10)
C6—N1—C2—S1	−178.13 (8)	C5—C6—C21—C22	30.48 (16)
C8—S1—C2—N3	168.96 (8)	C26—C21—C22—C23	2.90 (17)
C8—S1—C2—N1	−13.64 (10)	C6—C21—C22—C23	−176.82 (10)
N1—C2—N3—C4	2.13 (17)	C21—C22—C23—O3	179.04 (10)
S1—C2—N3—C4	179.18 (8)	C21—C22—C23—C24	−0.22 (16)
C2—N3—C4—N2	−179.21 (10)	O3—C23—C24—O4	−0.95 (14)
C2—N3—C4—C5	1.14 (15)	C22—C23—C24—O4	178.37 (10)
N2—C4—C5—C6	175.43 (11)	O3—C23—C24—C25	178.65 (10)
N3—C4—C5—C6	−4.93 (15)	C22—C23—C24—C25	−2.03 (16)
N2—C4—C5—C7	−8.73 (16)	O4—C24—C25—C26	−178.87 (10)
N3—C4—C5—C7	170.91 (10)	C23—C24—C25—C26	1.57 (17)
C2—N1—C6—C5	−2.96 (15)	C22—C21—C26—C25	−3.37 (17)
C2—N1—C6—C21	177.25 (9)	C6—C21—C26—C25	176.35 (10)
C7—C5—C6—N1	−169.69 (10)	C24—C25—C26—C21	1.15 (17)
C4—C5—C6—N1	5.90 (16)	O1—C9—O2—C10	1.39 (16)
C7—C5—C6—C21	10.08 (17)	C8—C9—O2—C10	−179.59 (9)
C4—C5—C6—C21	−174.33 (10)	C11—C10—O2—C9	−174.39 (10)
C2—S1—C8—C9	−69.51 (9)	C22—C23—O3—C27	−6.61 (16)
S1—C8—C9—O1	−36.23 (15)	C24—C23—O3—C27	172.68 (10)
S1—C8—C9—O2	144.75 (8)	C25—C24—O4—C28	10.82 (16)
N1—C6—C21—C26	30.55 (15)	C23—C24—O4—C28	−169.61 (10)
C5—C6—C21—C26	−149.23 (11)		

### Ethyl 2-{[4-amino-5-cyano-6-(3,4-dimethoxyphenyl)pyrimidin-2-yl]sulfanyl}acetate Hydrogen-bond geometry (Å, º)

**Table:**

*D*—H···*A*	*D*—H	H···*A*	*D*···*A*	*D*—H···*A*
N2—H021···N4^i^	0.862 (19)	2.201 (19)	3.0368 (15)	163.4 (16)
N2—H022···O1^ii^	0.879 (19)	2.139 (19)	2.9942 (13)	164.0 (16)
N2—H022···S1^ii^	0.879 (19)	2.973 (17)	3.4465 (11)	115.7 (13)
C8—H8*A*···N4^iii^	0.99	2.67	3.4693 (16)	138
C11—H11*A*···O3^iv^	0.98	2.52	3.1459 (15)	122
C27—H27*B*···S1^v^	0.98	2.95	3.6836 (12)	133

Symmetry codes: (i) −*x*+2, −*y*+2, −*z*+1; (ii) −*x*+2, *y*+1/2, −*z*+3/2; (iii) *x*, *y*−1, *z*; (iv) −*x*+1, *y*−1/2, −*z*+1/2; (v) −*x*+2, −*y*+1, −*z*+1.
